# MAP Kinase Hog1 Regulates Metabolic Changes Induced by Hyperosmotic Stress

**DOI:** 10.3389/fmicb.2016.00732

**Published:** 2016-05-18

**Authors:** Jiyoung Kim, Junsang Oh, Gi-Ho Sung

**Affiliations:** ^1^Institute for Bio-Medical Convergence, International St. Mary's Hospital and College of Medicine, Catholic Kwandong UniversityIncheon, Korea; ^2^Institute of Life Science and Biotechnology, Sungkyunkwan UniversitySuwon, Korea; ^3^College of Pharmacy, Chung-Ang UniversitySeoul, Korea

**Keywords:** Hog1/MAP kinase, hyperosmotic stress, ^1^H-nuclear magnetic resonance, *Beauveria bassiana*, *Saccharomyces cerevisiae*

Upon hyperosmotic stress, the High Osmolarity Glycerol (HOG) pathway is activated, resulting in phosphorylation of the stress-activated protein kinasei Hog1 (O'Rourke et al., 2002; Saito and Posas, 2012; Brewster and Gustin, 2014). The Hog1/mitogen-activated protein kinase-mediated signaling cascades are essential for sensing hyperosmotic stress and for transmitting these signals to the nucleus to modulate gene expression (O'Rourke et al., 2002; Saito and Posas, 2012; Brewster and Gustin, 2014). MAPK-mediated cascade pathways are composed of three serine/threonine protein kinases (MAPK kinase kinase, MAPK kinase, and MAPK). This signal transduction pathway is well-conserved in eukaryotes (O'Rourke et al., 2002; Saito and Posas, 2012; Brewster and Gustin, 2014).

Phosphorylated Hog1 stimulates expression of genes encoding enzymes involved in glycerol production and uptake (O'Rourke et al., 2002; Saito and Posas, 2012; Gomar-Alba et al., 2013; Lee et al., 2013; Babazadeh et al., 2014; Brewster and Gustin, 2014). The glycerol production starts with the reduction of the glycolytic intermediate di-hydroxyl-acetone phosphate (DHAP) to glycerol-3-phosphate (G3P) catalyzed by the NAD^+^-dependent glycerol-3-phospate dehydrogenase (GPD; Ansell et al., 1997; Pahlman et al., 2001; Valadi et al., 2004). Increased expression of *GPD1* enhances glycerol production under hyperosmotic stress (Albertyn et al., 1994; Rep et al., 1999). In addition, Hog1 appears to stimulate the 6-phosphofructo-2-kinase Pfk26, which produces fructose-2,6-diphosphate (F26DP), an allosteric activator of the glycolytic enzyme phosphofructokinase (Pfk1; Dihazi et al., 2004). Hog1 is rapidly phosphorylated upon curcumin treatment, an active polyphenol derived from the spice turmeric (Azad et al., 2014). *Trichosporonoides oedocephalis* is known to produce large amounts of polyols (erythritol and glycerol). *Tohog1* null mutation increased erythritol production and decreased glycerol production, respectively (Li et al., 2016). Therefore, Hog1 is considered to have an important role in the metabolic control, however little is known regarding the changes of metabolites induced by Hog1 from a metabolic perspective. Nuclear magnetic resonance (NMR) spectroscopy is a rapid method that requires minimal sample preparation (Beckonert et al., 2007; Kim et al., 2010). In addition, NMR is a useful technique for structure elucidation due to its various two-dimensional measurements, which makes NMR an ideal tool for the identification and quantification of metabolites (Beckonert et al., 2007; Kim et al., 2010).

## Materials and methods

### Materials

The *Beauveria bassiana* strain EFCC783 was grown on sterilized cellophane (Bio-Rad) in SDAY medium. The *Saccharomyces cerevisiae* wild-type (W303) and *hog1*Δ strains were grown on YPD medium.

### Complementation assay of *BbHog1* in *hog1*Δ strain

*BbHog1* was sub-cloned into the yeast expression vector *pD1218* (DNA2.0). Each *pD1218* construct was then transformed into the *hog1*Δ mutants. Positive transformants were selected in YPD medium containing geneticin (200 μg/ml), and confirmed using PCR. To evaluate the ability of BbHog1 to rescue cell growth under hyperosmotic stress, growth was compared on YPD versus YPD containing 0.8 M NaCl. Plates were incubated for 24 h at 30°C.

### Western blot analysis

Yeast cells were treated with 0.8 M NaCl for 20 min. Their protein extracts (30 μg) were separated by SDS-PAGE using 10% polyacrylamide gel and analyzed by Western blotting using the anti-p-Hog1/p38 or anti-Hog1/p38 antibodies (Cell Signaling) and a secondary horseradish peroxidase-conjugated anti-IgG antibody (Sigma-Aldrich). Enhanced chemiluminescent reagent (Bio-Rad) was used to detect the proteins.

### Proton nuclear magnetic resonance (^1^H-NMR)

Yeast cells were treated with 0.8 M NaCl for 40 min, and then washed following the rapid quenching procedure [60% MeOH and 0.85% AMBIC (pH 7.4)]. The quenched cells were dissolved in 90 mM phosphate buffer in D_2_O, containing 0.01 % TSP as an internal standard. After cell lysis, the supernatants were clarified by centrifuging at 15,000 rpm for 30 min at 4°C, filtered by 3 kDa cutoff filters (Millipore), and collected into 1.5 ml tubes. Then, 600 μl of each filtered extract was loaded into 5 mm NMR tubes. ^1^H-NMR spectra were acquired at 300 K on a 600.13-MHz Bruker Avance Spectrometer (Bruker) using the standard zgpr pulse sequence (Kim et al., 2010). In total, 128 transients were gathered into 32 K data points with a relaxation delay of 2 s, an acquisition time of 1.70 s per scan, and a spectra width of 12.0 ppm. The NMR spectra were analyzed using Chenomx NMR suite software v8.1 (Fan, 1996).

### ^1^H-NMR raw data processing

^1^H-NMR raw files (.fid) produced by a 600.13-MHz Bruker Advance spectrometer were imported and analyzed using Chenomx NMR suite software v8.1 (Chenomx). The Chenomx NMR suite software v8.1 (Chenomx) converted ^1^H-NMR raw files (.fid) to Chenomx file format (.cnx). Each spectral intensity dataset was normalized to the assigned chemical compounds according to total sum of the spectral regions, converted to Microsoft Office format, and imported into Web-based MetaboAnalyst v3.0 software to carry out our normalization, scaling, and multivariate analysis. Multivariate statistical analyses were performed by one-way ANOVA using PASW Statistics 22 software (IBM) followed by a Tukey's significant difference test. Significance was determined with a *p*-value threshold (<0.05). Metabolites levels were normalized using the log_2_ function and then, mean centering and Pareto scaling was applied for all PCA and Heatmaps by MetaboAnalyst v3.0. The hierarchical clustering analysis (HCA) also shows clustering based on the replicate values of averaged appropriate and Ward's method on Euclidean distance matrix.

## Results

*Beauveria bassiana* is an important entomopathogenic fungus with wide application for biological control of harmful insects in the world (Posada et al., 2004). We isolated a gene for the Hog1 through searching the genome database of *B. bassiana*. The predicted molecular weight of BbHog1 was 40.9 kDa and its *p*I was 5.45, respectively. Our initial attempt at producing *BbHog1* knockout mutants was unsuccessful. Moreover, we failed to get the *Bbhog1*Δ (Zhang et al., 2009). Alternatively, we employed complementation strategies using *S. cerevisiae hog1*Δ mutant. The *hog1*Δ strain was transformed with plasmids driving the expression of *BbHog1*. The viability of transformed yeast cells was then determined in medium containing 0.8 M NaCl. *BbHog1* successfully complemented the *hog1*Δ phenotype under hyperosmotic stress (Figure S1). In addition, BbHog1 can be phosphorylated in yeast under hyperosmotic stress (Figure S2). Therefore, our results indicate that BbHog1 is functional in *S. cerevisiae*.

**Figure 1:**
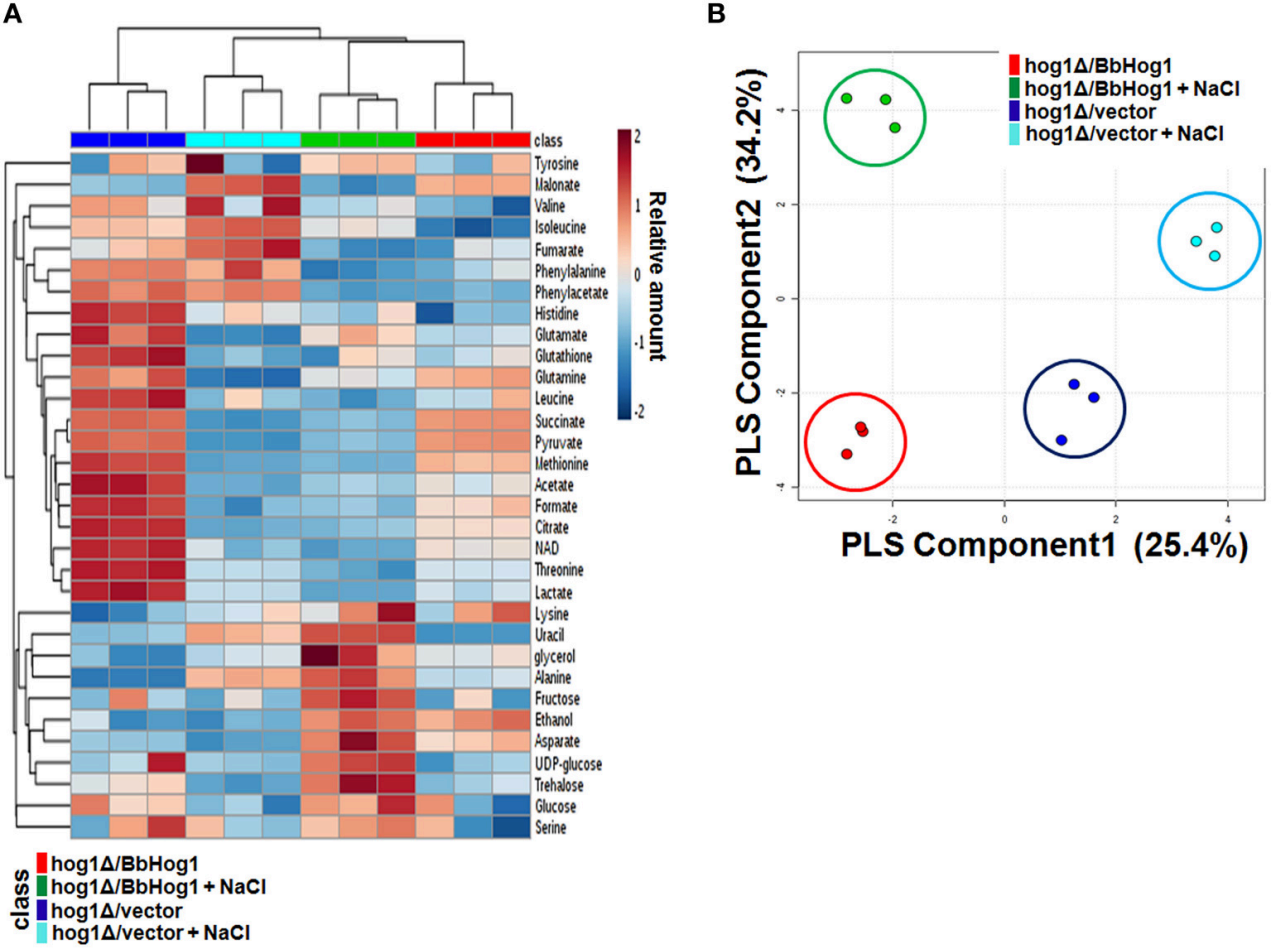
**Heatmap of main metabolite variations (A) and PLS-DA score plot (B) in *hog1*Δ*/vector* and *hog1*Δ*/BbHog1* under hyperosmotic stress**.

To explore metabolic changes during hyperosmotic stress response in *hog1*Δ*/vector* and *hog1*Δ*/BbHog1*, we profiled the metabolites in each strain using ^1^H-NMR spectroscopy. Chemical shifts of signals were assigned to the metabolites in the area of amino acids, organic acids, carbohydrates, and nucleotide derivatives. According to the ^1^H-NMR spectra, we identified 32 metabolites from the whole yeast extracts with chemical shifts and coupling patterns (Figures S3, S4). The main variations are summarized in the form of a heatmap shown in Figure 1A and Figure S5. ^1^H-NMR spectra of *hog1*Δ*/vector* and *hog1*Δ*/BbHog1* showed differential resonance spectra under hyperosmotic stress.

Multivariate data analysis is used to identify differences or similarities among the samples. Each point of the score plot in multivariate analysis represents an individual sample. Grouping and outliers of samples can be easily observed in a score plot. Partial least squares-discriminant analysis (PLS-DA) was used to investigate intrinsic variation in ^1^H-NMR data. In the PLS-DA score plot, two principal components, PC1 and PC2, were calculated with the R^2^Y and Q^2^Y parameters of 0.990 and 0.955 (Figure 1B). As shown in Figure 1B, the PLS-DA score plot of ^1^H-NMR spectra showed a clear separation between *hog1*Δ*/vector* and *hog1*Δ*/BbHog1* under hyperosmotic stress.

**Figure 2:**
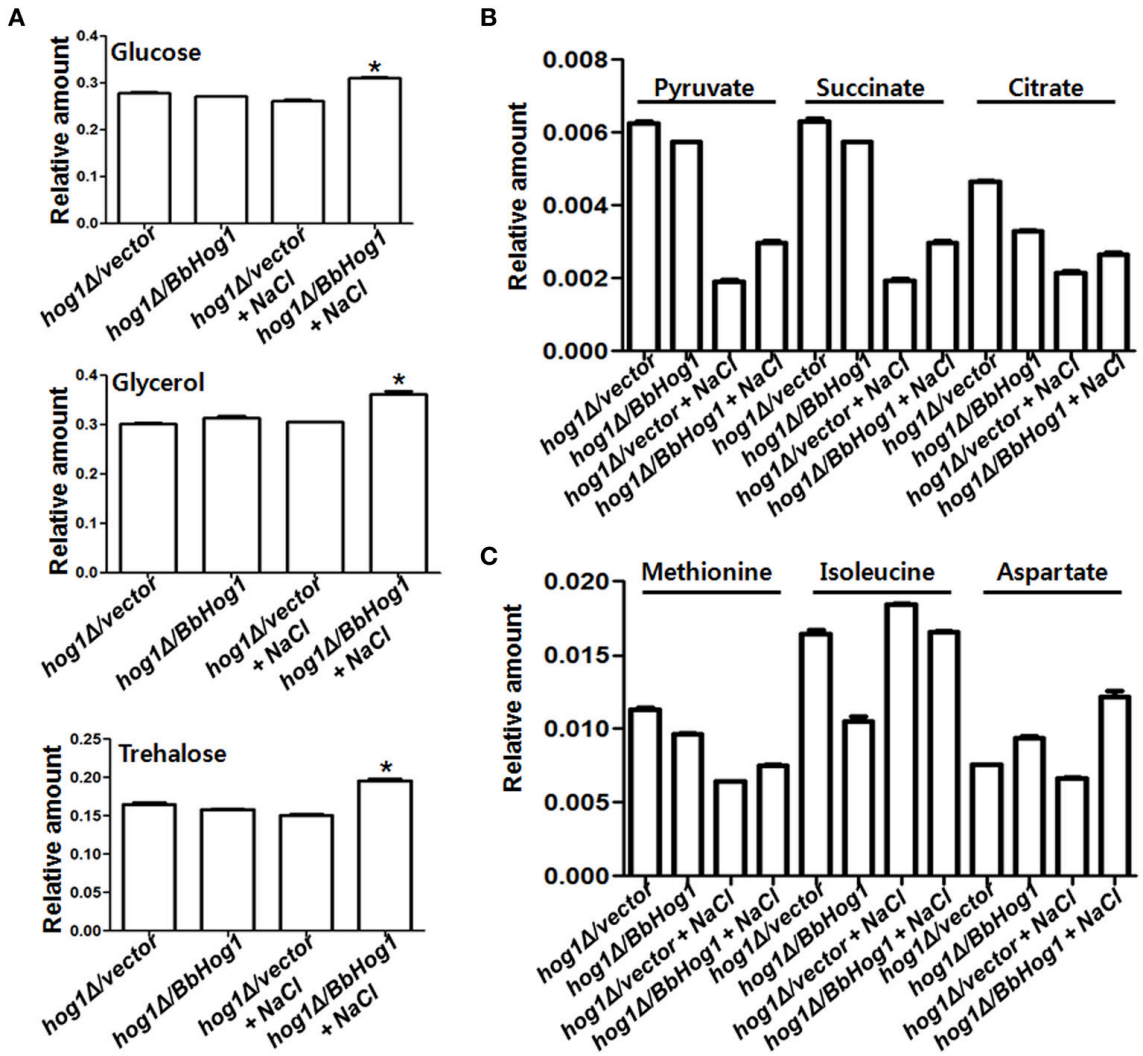
**Hog1 regulates cellular concentrations of a variety of metabolites under hyperosmotic stress**. Cellular concentrations of glucose, glycerol, and trehalose **(A)**, and succinate, citrate and pyruvate **(B)**, and Met, Ile, and Asp **(C)** are differentially regulated in *hog1*Δ*/vector* and *hog1*Δ*/BbHog1* under hyperosmotic stress. Experimental values are means of three independent experiments with standard deviation (Student's *t*-test, ^*^p < 0.01).

Glycerol and trehalose function as representative osmolytes in order to cope with changes in hyperosmotic stress (Hounsa et al., 1998; Hohmann, 2002). According to ^1^H-NMR data, cellular concentrations of glucose, glycerol (Albertyn et al., 1994), and trehalose were increased under hyperosmotic stress in *hog1*Δ*/BbHog1*, but not in *hog1*Δ*/vector* (Figure 2A). Glucose is the main energy source in yeast. Maintenance of ATP, the main energy source, homeostasis is critical for all cells. In response to hyperosmotic stress, the higher energy demands required for hyperosmotic stress tolerance are allotted by a rapid alteration in cellular ATP metabolism (Olz et al., 1993; Oren, 1999). Therefore, the increased levels of glucose induced by Hog1 might be required to satisfy the higher energy requirements, as well as increased production of osmolytes such as glycerol and trehalose during hyperosmotic stress signaling.

In general, glucose inhibits the expression of genes involved in respiration (Hardie et al., 1998). In addition, multiple stress environments suppress the expression of genes associated with the tricarboxylic acid (TCA) cycle (Diano et al., 2009). Through, ^1^H-NMR data, we investigated whether Hog1 regulates cellular concentrations of TCA cycle intermediates under hyperosmotic stress. As shown in Figure 2B, the cellular concentrations of succinate and citrate were slightly reduced in *hog1*Δ*/BbHog1* under normal conditions. Succinate and citrate were largely reduced in *hog1*Δ*/vector* in response to hyperosmotic stress, but these reductions were significantly reduced in *hog1*Δ*/BbHog1*. Pyruvate can be made from glucose through glycolysis, or converted back to glucose via gluconeogenesis. Under hyperosmotic stress, the cellular level of pyruvate had a similar pattern with those of succinate and citrate (Figure 2B). Therefore, our results suggest that hyperosmotic stress lead to reductions in pyruvate and TCA cycle intermediates, and that Hog1 plays a role in mediating these metabolic changes under hyperosmotic stress.

Hyperosmotic stress alters cellular concentration of amino acids as the cell attempts to survive against hyperosmotic stress (Kovács et al., 2012). As shown in Figure 2C, the cellular concentration of methionine (Met) was largely reduced in *hog1*Δ*/vector* in response to hyperosmotic stress, but these reductions were significantly reduced in *hog1*Δ*/BbHog1*. Cellular concentration of isoleucine (Ile) was largely reduced under normal conditions in *hog1*Δ*/BbHog1*, but not in *hog1*Δ*/vector*. In case of aspartate (Asp), its cellular concentration was increased under hyperosmotic stress in *hog1*Δ*/BbHog1*, not in *hog1*Δ*/vector* (Figure 2C). Therefore, our results suggest that hyperosmotic stress induces metabolic changes of amino acids such as Met, Ile, and Asp, and Hog1 plays a role in these metabolic regulations during hyperosmotic stress.

In summary, this work represents that MAP kinase Hog1 regulates cellular concentrations of a variety of metabolites during hyperosmotic stress signaling.

## Author contributions

JK and GS designed the study. JK and JO performed the experiments. JK, JO, and GS analyzed data. JK and GS wrote the paper.

## Data access

Our ^1^H-NMR source files were deposited in figshare (https://dx.doi.org/10.6084/m9.figshare.2066538.v1).

### Conflict of interest statement

The authors declare that the research was conducted in the absence of any commercial or financial relationships that could be construed as a potential conflict of interest.

## Supplementary material

Figure S1 ***BbHog1* is functional in *S. cerevisiae***. Yeast cells were spotted on YPD plates and YPD plates containing 0.8 M NaCl at 30°C for 24 h.

Figure S2 **BbHog1 phosphorylation under hyperosmotic stress**. Yeast cells were treated with 0.8 M NaCl for 20 min, and their protein extracts were then subjected to Western blot analysis with anti-p-Hog1 or anti-Hog1 antibodies.

Figure S3 **^1^H-NMR spectra of the metabolites of *S. cerevisiae***.

Figure S4 **Chemical shifts of metabolite compounds according to ^1^H-NMR**.

Figure S5 **Relative metabolite contents of *S. cerevisiae* according to ^1^H-NMR**. Experimental values are the means of three independent experiments with standard deviation.
