# Supplementary figures and images for: MAP Kinase Hog1 Regulates Metabolic Changes Induced by Hyperosmotic Stress
Source: Front Microbiol. 2016 May 18;7:732. doi: 10.3389/fmicb.2016.00732 (PMC4870262; doi:10.3389/fmicb.2016.00732)

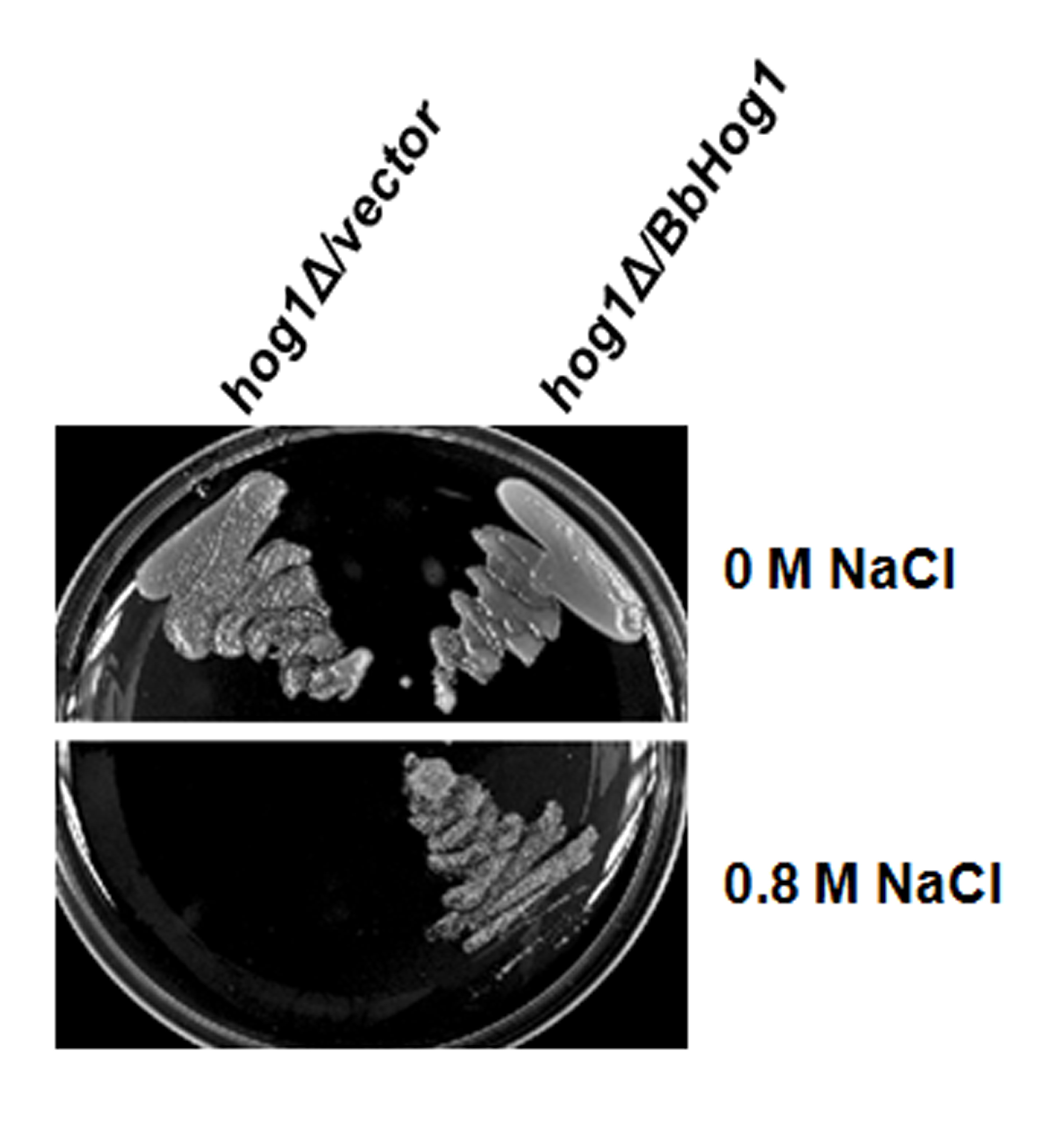

Supplement: Figure S1 — BbHog1 is functional in S. cerevisiae. Yeast cells were spotted on YPD plates and YPD plates containing 0.8 M NaCl at 30°C for 24 h. [file Image1.JPEG]

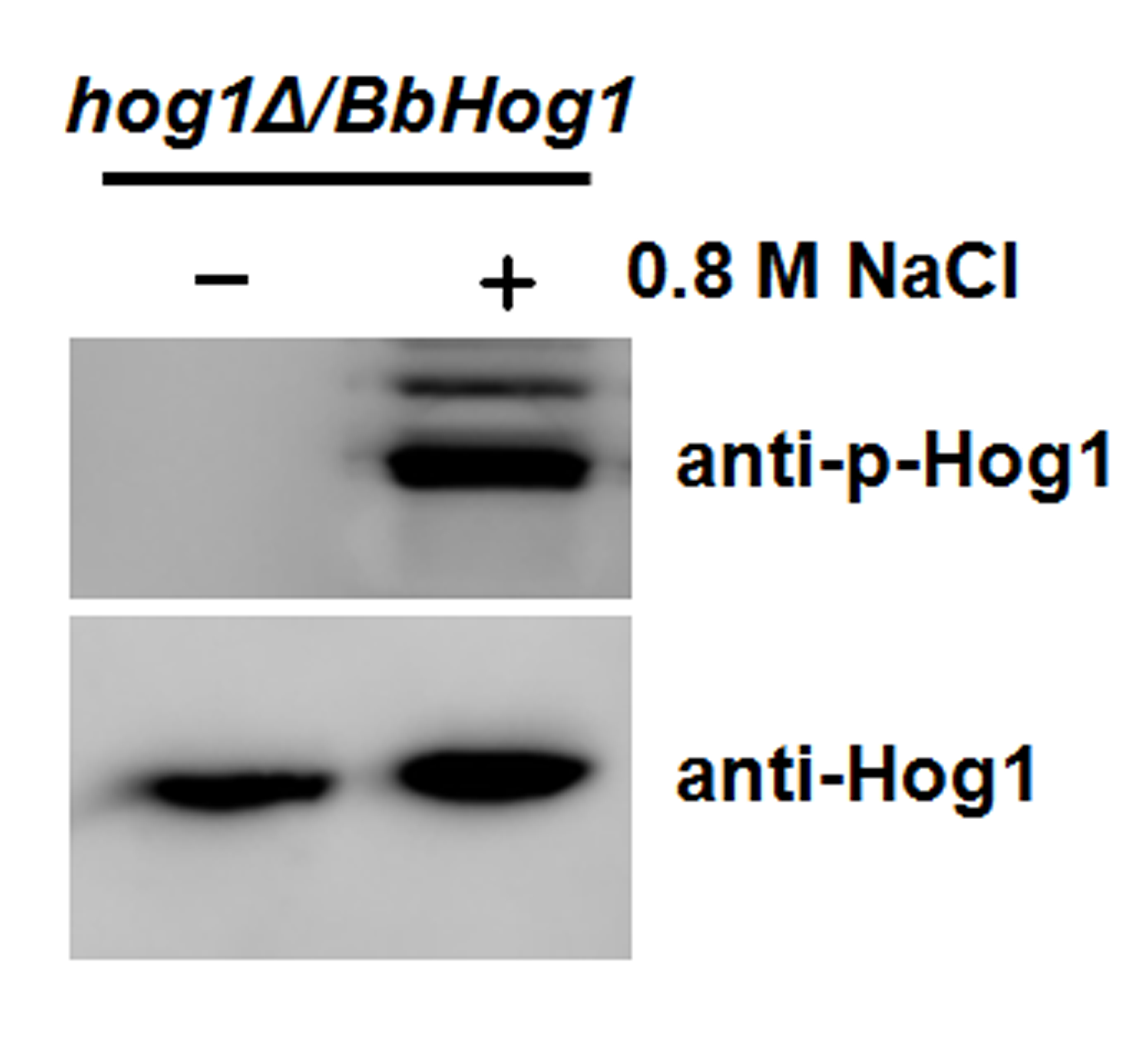

Supplement: Figure S2 — BbHog1 phosphorylation under hyperosmotic stress. Yeast cells were treated with 0.8 M NaCl for 20 min, and their protein extracts were then subjected to Western blot analysis with anti-p-Hog1 or anti-Hog1 antibodies. [file Image2.JPEG]

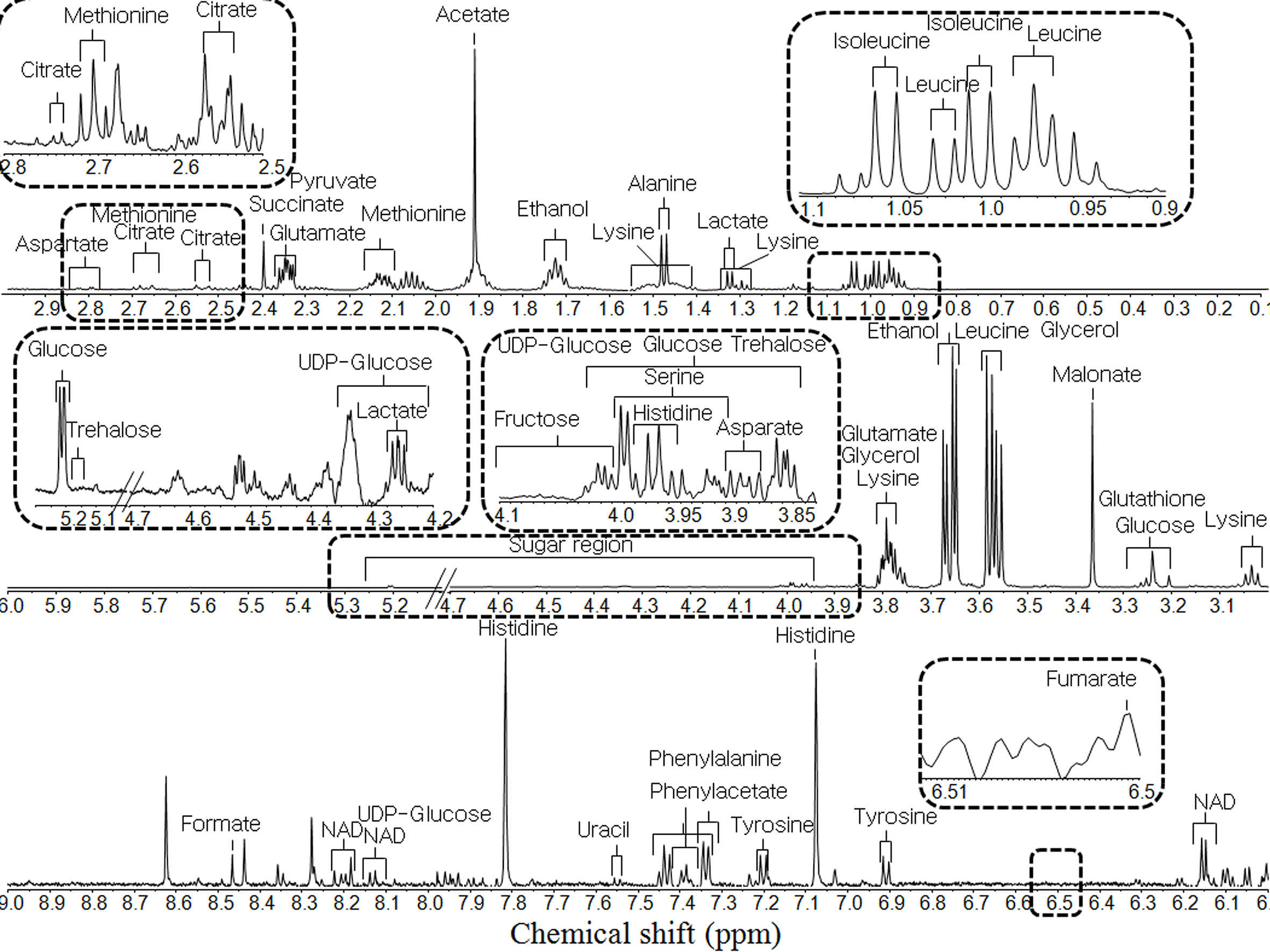

Supplement: Figure S3 — 1H-NMR spectra of the metabolites of S. cerevisiae. [file Image3.JPEG]

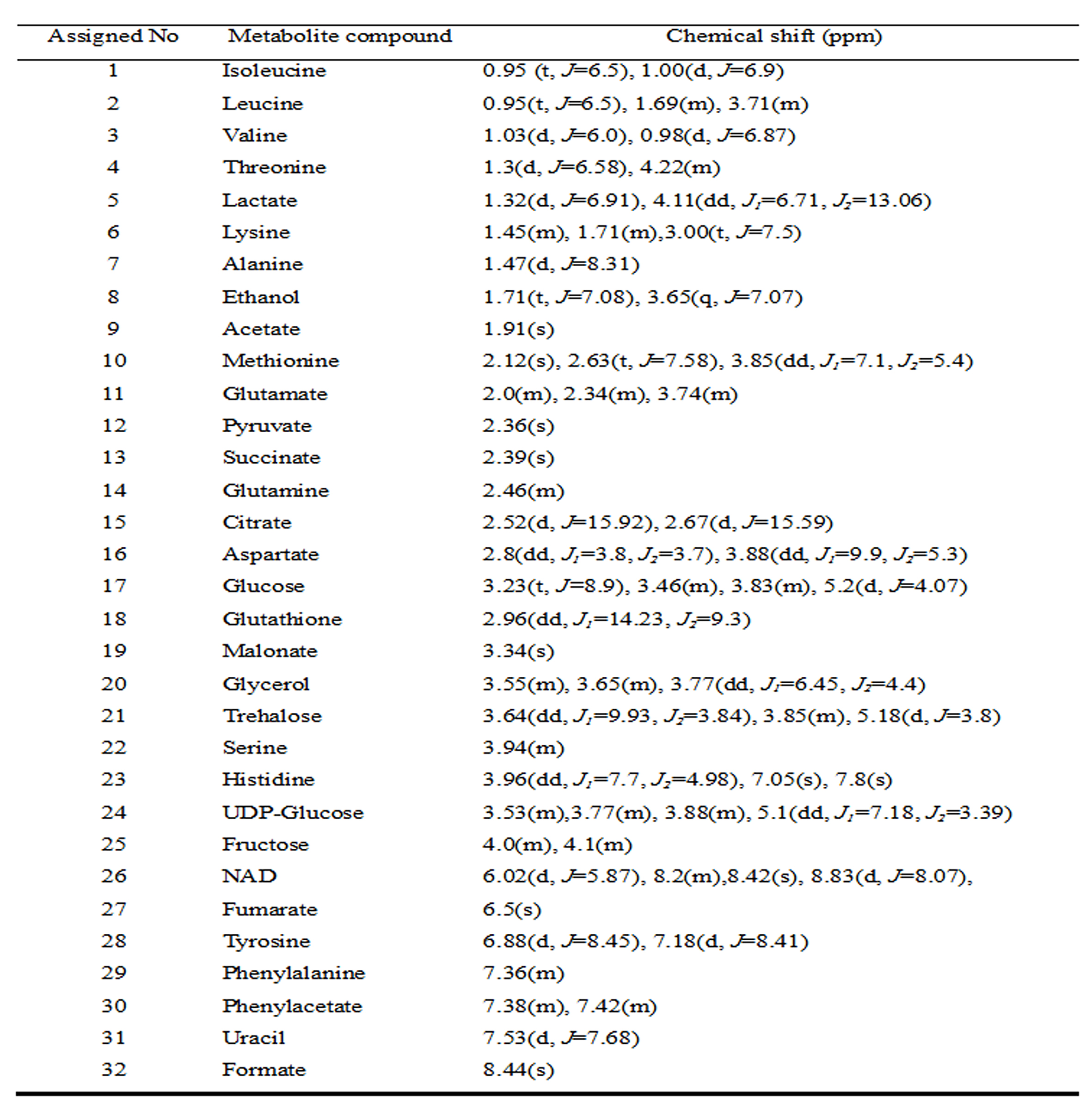

Supplement: Figure S4 — Chemical shifts of metabolite compounds according to 1H-NMR. [file Image4.JPEG]

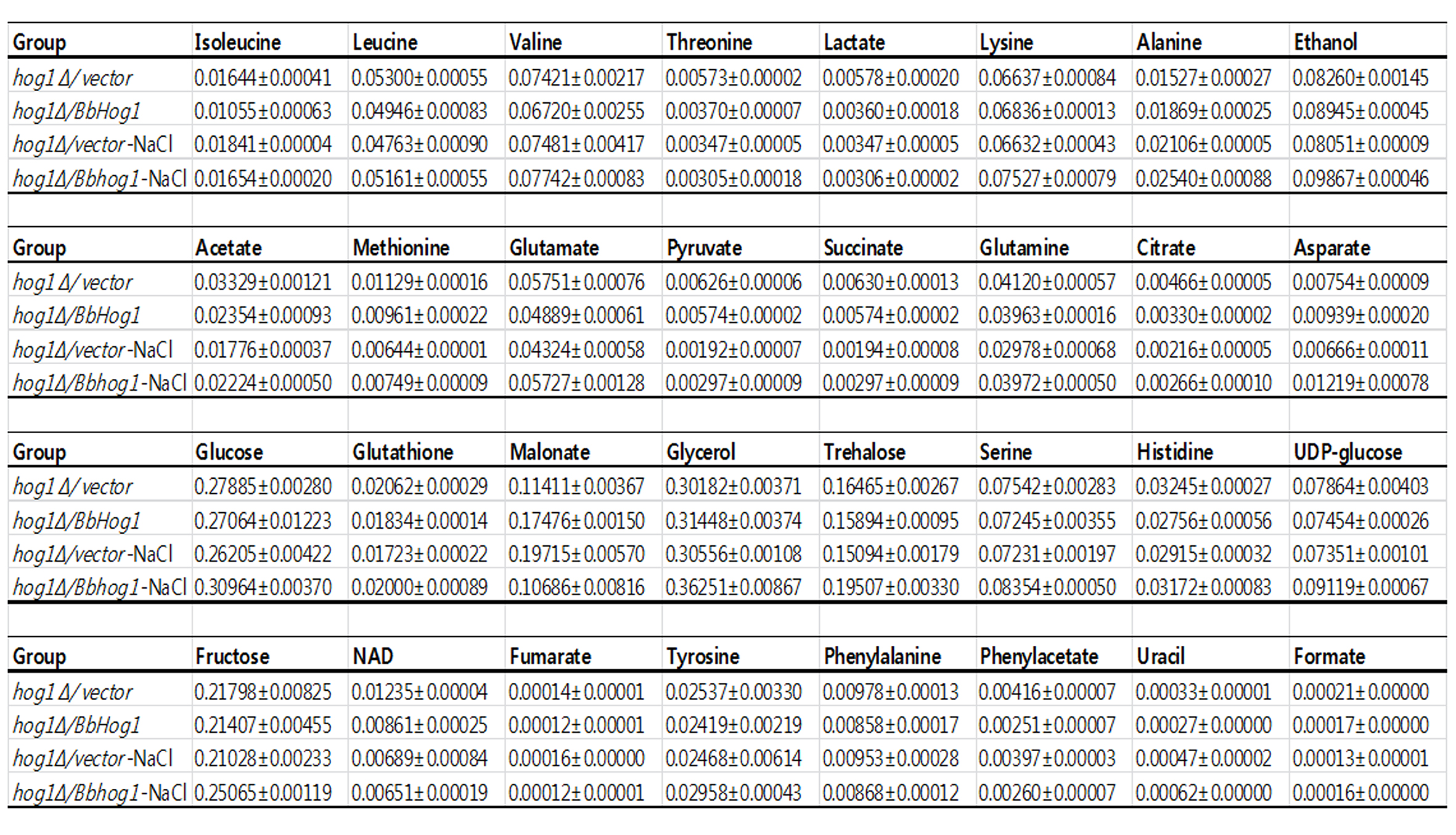

Supplement: Figure S5 — Relative metabolite contents of S. cerevisiae according to 1H-NMR. Experimental values are the means of three independent experiments with standard deviation. [file Image5.JPEG]
